# Metformin Reduces the Incidence of Sensorineural Hearing Loss in Patients With Type 2 Diabetes Mellitus: A Retrospective Chart Review

**DOI:** 10.7759/cureus.30406

**Published:** 2022-10-17

**Authors:** Toru Miwa, Tomoko Kita, Taro Yamaguchi, Tatsunori Sakamoto

**Affiliations:** 1 Otolaryngology, Osaka Metropolitan University, Osaka, JPN; 2 Otolaryngology-Head and Neck Surgery, Kyoto University, Kyoto, JPN; 3 Laboratory of Pharmacology, Setsunan University, Osaka, JPN; 4 Otolaryngology, Faculty of Medicine, Shimane University, Shimane, JPN

**Keywords:** metformin, multivariate logistic regression analysis, propensity score matching (psm), diabetes mellitus type 2, sensorineural (sn) hearing loss

## Abstract

Introduction and objectives

Acquired sensorineural hearing loss (SNHL) has become a critical societal issue in recent years. SNHL is considered a risk factor for type 2 diabetes mellitus (T2DM). Metformin is commonly used to treat T2DM. However, its effects on SNHL have not been reported yet. Hence, this study aimed to evaluate the association between the use of metformin and SNHL incidence.

Patients and methods

In this retrospective matched-cohort study, the medical records of 1219 patients with T2DM aged >18 years from our hospital’s inpatient database from January 1, 2012, to December 31, 2019, were examined, and matched cohorts were generated (76 patients receiving metformin and 76 not receiving metformin). A multivariable logistic regression analysis was performed to investigate the factors influencing the incidence of SNHL.

Results

After adjustment by propensity matching, multivariable logistic regression analysis revealed that the non-use of metformin increased the risk of developing SNHL (odds ratio, 0.26; 95% confidence interval, 0.07-0.93; p = 0.03).

Conclusions

This study demonstrated an association between the use of metformin and a reduced incidence of SNHL among patients with T2DM.

## Introduction

Acquired sensorineural hearing loss (SNHL) has recently become a critical societal issue [1,2]. The incidence of SNHL has increased by 1.5% annually [3,4], and most types of SNHL may result in permanent hearing disability. SNHL occurs due to damage to the inner ear, the nerve running from the ear to the brain (auditory nerve), or the brain [1]. Although several mechanisms have been proposed, including inflammation, oxidative stress, and blood flow disturbances in the inner ear, the etiology of SNHL remains unclear [2,5]. The pathophysiology of SNHL has not been fully elucidated, and preventing its onset remains challenging. One factor complicating inner ear therapy is the difficulty of delivering drugs to the inner ear after systemic administration. Multiple clinical trials have been conducted utilizing topical administration [6]. However, it is not commonly used clinically, and no such drug is currently available on the market. Therefore, drug repositioning has emerged as an efficient drug discovery method that reduces the need for long-term clinical trials [7].

SNHL is a risk factor for type 2 diabetes mellitus (T2DM) [8,9]. Lin et al. reported that diabetes mellitus (DM) was significantly associated with an increased risk of developing idiopathic sudden SNHL, with an adjusted hazard ratio of 1.592 and an incidence rate that is 1.54-fold higher in the DM group than that in the non-DM group [10]. Moreover, metabolic syndromes such as DM have been associated with an increased risk of developing idiopathic sudden SNHL in large-scale studies [11,12]. Krishnappa et al. reported that DM was significantly associated with an increased risk of developing age-related SNHL [13]. Therefore, it is necessary to evaluate whether medications used in the treatment of DM have a simultaneous protective effect against SNHL [14]. We focused on metformin, an anti-diabetes biguanide drug, as a candidate for the prevention of deafness because of its efficiency in reaching the inner ear [15]. Few reports have indicated the usefulness of metformin for SNHL [14,16].

We hypothesized that metformin has a preventive effect on the incidence of SNHL. We aimed to evaluate the association between the use of metformin and SNHL incidence.

## Materials and methods

Study design

**Figure 1:**
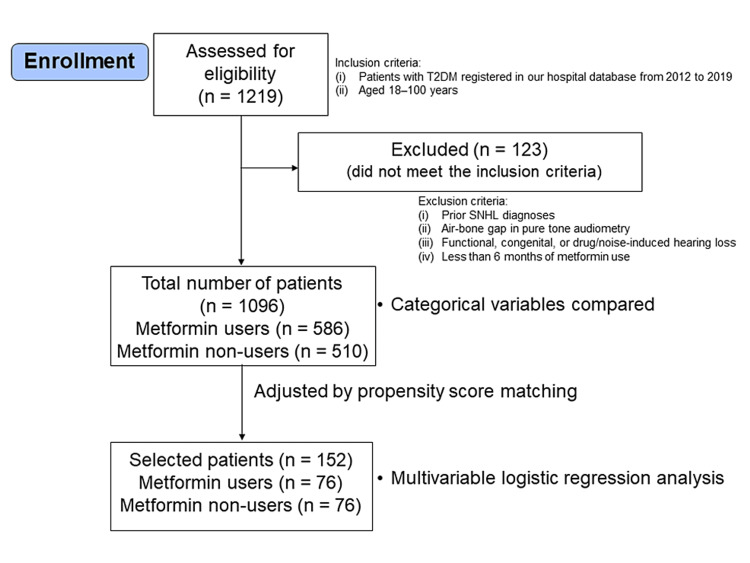
Flowchart of the retrospective study. T2DM: type 2 diabetes mellitus; SNHL: sensorineural hearing loss.

This study used a retrospective cohort design (Figure 1). Data were acquired from our hospital database, which included pure-tone audiometry data (hospitalization longitudinal database). The inclusion criteria were as follows: (i) patients with T2DM registered in our hospital database from January 1, 2012, to December 31, 2019, and (ii) aged 18-100 years. The study enrolled 1219 patients with T2DM regardless of metformin use, of which 123 patients complained of hearing loss prior to the study period. SNHL was diagnosed according to the criteria defined by the International Statistical Classification of Diseases and Related Health Problems, 10th Revision (ICD-10) codes. In brief, SNHL was defined as a unilateral or bilateral hearing loss of at least 30 dB in three sequential frequencies in the standard pure-tone audiogram that is not conductive [17,18]. The exclusion criteria included (i) prior SNHL diagnoses; (ii) an air-bone gap greater than 15 dB in pure-tone audiometry; (iii) functional, congenital, or drug/noise-induced hearing loss; and (iv) less than six months of metformin use. A total of 123 patients were excluded.

The primary endpoint in this study was the incidence of SNHL among diabetics, metformin users. We compared the categorical variables between metformin users and non-users to identify the confounding factors. After propensity matching with confounding factors (Appendix 1, Table 2), multivariable logistic regression analysis was performed to evaluate the incidence of SNHL. Explanatory variables that could affect the risk of SNHL (i.e., age, sex, metformin use, medical comorbidities, catastrophic illness, and history of disease) were considered potential confounding factors.

Ethics approval

The Institutional Review Board of our institution approved this study (Approval number: 2001005). The requirement for written informed consent to access data from our hospital database was waived because of the retrospective nature of the study.

Evaluation of hematological parameters, comorbidities, and history of the disease

The comorbidities assessed were hypertension (ICD10 I10.15); hyperlipidemia (ICD10 E78.5); cerebrovascular diseases, such as stroke (ICD10 I60-69); cardiovascular diseases, such as ischemic heart disease (ICD10 I20-25); rheumatic arthritis (ICD10 M06); hepatopathy (ICD10 K70-77); and thyroid dysfunction (ICD10 E00-07). Patient histories were evaluated for conditions such as tumors (ICD10 C00-97), dizziness (ICD10 R42), facial palsy (FP) (ICD10 G51), anxiety (ICD10 F41), depression (ICD10 F32), and insomnia (ICD10 G47). Hematological tests for hemoglobin A1c (HbA1c), blood urea nitrogen, and creatinine were performed in all patients. The estimated glomerular filtration rate (eGFR) was calculated using the Cockcroft-Gault formula.

Sample size analysis

The required sample size was estimated based on a threshold response rate of 35%, an expected response rate of 50%, a power of 80%, and an alpha value of 0.1 (one-sided) using the binomial test. Given a 2% ineligibility rate, the target sample size was determined to be at least 66 patients.

Data analysis

For comparison of the metformin user and non-user groups, differences in categorical variables were analyzed using Fisher’s exact test, and continuous variables were analyzed using an unpaired Student’s t-test.

The incidence of SNHL was determined using multivariate logistic regression, and the results are presented as odds ratios (ORs) with 95% confidence intervals (CIs). The outcome variable was the incidence of SNHL. The model was created after confirming the variance inflation factor. Explanatory variances were selected according to the Akaike information criterion. Missing values were input using the random forest method. No outliers were detected in the analysis.

A two-tailed p-value <0.05 was considered statistically significant. The evaluation was determined to be “not applicable” if the calculated sample size following data collection was insufficient for statistical analysis. All statistical analyses were performed using easy R (EZR) (Saitama Medical Center, Jichi Medical University, Saitama, Japan), which is a graphical user interface for R (The R Foundation for Statistical Computing, Vienna, Austria), and GraphPad Prism version 8.0.0 for Windows (GraphPad Software, San Diego, CA). The researchers were blinded to the study groups during data analysis.

## Results

Comparison of categorical variables between metformin use and non-use

Among the 1096 patients with T2DM, 586 (53.5%) used metformin and 510 (46.5%) did not. Significant between-group differences were observed in terms of age (63.74 vs. 69.15 years, p < 0.001), eGFR (67.17 vs. 60.45 mL/min/1.73 m^2^, p < 0.001), HbA1c level (8.09 vs. 7.87%, p = 0.02), cardiovascular disease (positive rate: 41.81 vs. 53.33%, p = 0.002), thyroid dysfunction (positive rate: 19.11 vs. 27.45%, p = 0.001), tumors (positive rate: 28.50 vs. 34.31%, p = 0.04), FP (positive rate: 10.07 vs. 9.22%, p = 0.01), and anxiety (positive rate: 12.46 vs. 19.61%, p = 0.001) (Appendix 1, Table 2).

Multivariable logistic regression analysis

After propensity matching with confounding factors, matched cohorts of 76 patients using and not using metformin were created. Among the 152 patients with T2DM, multivariate logistic regression analysis revealed that the incidence of SNHL in patients with T2DM was significantly associated with metformin use (OR, 0.26; 95% CI, 0.07-0.93; p = 0.03) and eGFR (OR, 0.95; 95% CI, 0.92-0.99; p = 0.03) (Table 1).

**Table 1:** Logistic regression analysis for the propensity score-matched patient cohort. CI: confidence interval, eGFR: estimated glomerular filtration rate, HT: hypertension. *p < 0.05.

Explanatory variable	Odds ratio	95% CI	p-value
Metformin	0.26	0.07–0.93	0.03*
eGFR	0.95	0.92–0.99	0.03*
HT	4.83	0.98–3.60	0.05
Dizziness	6.08	0.86–42.60	0.06

## Discussion

This retrospective, propensity score-matched cohort study explored the association between the incidence of SNHL and metformin use in patients with T2DM. Multivariable logistic regression analysis revealed that metformin use is a preventive factor affecting the incidence of SNHL. We found that metformin use may prevent the progression of hearing loss in patients with T2DM as well as provide novel evidence regarding the relationship between metformin use and the incidence of SNHL in patients with T2DM.

Metformin is hydrophilic, positively charged biguanide compound that acts as an insulin receptor sensitizer and anti-hyperglycemic agent. It exerts anti-inflammatory, antiangiogenic, proapoptotic, and antioxidant effects by stimulating the activation of adenosine monophosphate-activated protein kinase (AMPK)/extracellular signal-regulated kinase 1 (ERK1)/extracellular signal-regulated kinase 2 (ERK2), which reduces vascular inflammation and protects the endothelium using nitric oxide synthase [19,20]. Metformin reportedly decreases the chronic accumulation of reactive oxygen species (ROS) via ROS scavenging and prevention of ROS production, exerts protective effects against cerebral and cardiovascular diseases, and reduces the risk of stroke in patients with DM [21-23]. Our data revealed a significantly reduced percentage of cardiovascular disease in all patients with DM receiving metformin. Cardiovascular diseases are associated with a high risk of SNHL [24]. The mechanisms underlying the effects of metformin use on the risk of SNHL remain unclear. In vitro and in vivo evidence suggests that metformin protects against hair cell death caused by gentamicin-induced, cisplatin-induced, radiation-induced, or noise-induced ototoxicity [15,25]. Föller et al. demonstrated that the AMPK pathway participates in signaling cascades that protect the inner ear from damage following acoustic overstimulation [26]. These findings support the beneficial effects of metformin use in decreasing the risk of idiopathic sudden SNHL in patients with DM. Additionally, metformin modulates aging-associated inflammation and cellular degeneration and dysfunction by regulating nuclear factor-κB [16,27]. Thus, the results of basic research suggest that the use of metformin may be feasible for inner ear therapy in patients with SNHL, even in those without T2DM.

Our study has some limitations. First, information on the precise dose and duration of metformin use was not available. Second, the sample size of patients with SNHL was small. Third, this population-based study could not clarify the mechanisms underlying the association between metformin use and the incidence of SNHL in patients with T2DM. Further research is warranted to investigate these issues and promote the generalizability of the results.

## Conclusions

This study demonstrated an association between the use of metformin and a reduced SNHL incidence among patients with T2DM. We suggest that metformin use may prevent the progression of hearing loss in patients with T2DM as well as provide novel evidence regarding the relationship between metformin use and the incidence of SNHL in patients with T2DM. Further research regarding to understanding the mechanism is warranted to investigate these issues and promote the generalizability of the results.

## Appendices

Appendix 1

**Table 2:** Comparisons between metformin users and non-users. eGFR: estimated glomerular filtration rate, HbA1c: hemoglobin A1c, HL: hyperlipidemia, HT: hypertension, Met+: metformin users, Met-: metformin non-users, RA: rheumatic arthritis, FP: facial palsy. *p < 0.05, **p < 0.01, ***p < 0.001.

Patient characteristics	Met+	Met-	p-value
Number	586	510	
Age (mean, years)	63.74	69.15	<0.001***
Sex (female/male)	0.55	0.63	0.31
eGFR (mean, mL/min)	67.17	60.45	<0.001***
HbA1c (mean, %)	8.09	7.87	0.02*
HT (Yes/No, %)	72.70	70.00	0.34
HL (Yes/No, %)	78.16	76.27	0.47
Cerebrovascular disease (Yes/No, %)	32.76	36.08	0.25
Cardiovascular disease (Yes/No, %)	41.81	53.33	0.002**
RA (Yes/No, %)	5.12	7.65	0.10
Hepatopathy (Yes/No, %)	39.25	37.45	0.57
Thyroid dysfunction (Yes/No, %)	19.11	27.45	0.001**
Tumors (Yes/No, %)	28.50	34.31	0.04*
Dizziness (Yes/No, %)	14.68	16.47	0.45
FP (Yes/No, %)	10.07	9.22	0.01*
Anxiety (Yes/No, %)	12.46	19.61	0.001**
Depression (Yes/No, %)	9.56	12.35	0.14
Insomnia (Yes/No, %)	34.98	35.88	0.80
